# Climate Change Adaptation Methods for Public Health Prevention in Australia: an Integrative Review

**DOI:** 10.1007/s40572-023-00422-7

**Published:** 2024-01-15

**Authors:** Tony G. Walter, Lisa K. Bricknell, Robyn G. Preston, Elise G. C. Crawford

**Affiliations:** 1 School of Health, Medical and Applied Sciences, Central Queensland University, 554-700 Yaamba Road, Norman Gardens, 4701 Australia; 2 School of Health, Medical and Applied Sciences, Central Queensland University, 538 Flinders Street, Townsville, QLD 4810 Australia

**Keywords:** Climate change adaptation, Public health, Risk management, Vulnerability, Resilience, Health system

## Abstract

**Purpose of Review:**

Climate change poses a serious threat to human health and well-being. Australia is not immune to the public health impacts and continues to be underprepared, putting the population health at risk. However, there is a dearth in knowledge about how the Australian public health system will address the impacts of climate change.

**Recent Findings:**

This integrative review synthesises tools, frameworks, and guidance material suitable for climate change adaptation from a preventive public health perspective. The literature search was conducted in electronic databases MEDLINE, PubMed, CINAHL, and Web of Science. Of 4507 articles identified, 19 articles met the inclusion criteria that focused on operational methods in public health and excluded the clinical context and reactive disaster response approaches.

**Summary:**

This review revealed that Australia is ill-prepared to manage climate change adverse health impacts due to ineffective adaptation strategies. The review highlights that Australia urgently requires effective adaptation strategies such as undertaking a National Adaptation Plan process and an improved understanding in managing complex health risks. Taking this action will strengthen the public health system and build health resilience especially for vulnerable populations. These findings will help understand and develop of the necessary adaptive strategies in Australia.

**Supplementary Information:**

The online version contains supplementary material available at 10.1007/s40572-023-00422-7.

## Introduction

Climate change continues to pose a serious threat to human and planetary health [1]. The Intergovernmental Panel on Climate Change (IPCC) warns that the window of opportunity to maintain a liveable sustainable future is narrowing [1]. Human-induced changes are introducing new pressures on human health and a burdening the health system, exacerbating existing challenges [2, 3]. For instance, Australia has suffered recently from two unprecedented national climate catastrophes: firstly, devastating bushfires followed by torrential flooding that resulted in loss of lives and negative impacts on the population’s wellbeing [4, 5]. Climate change has multiple and complex pathways that influence human health conditions having both direct and indirect effects [3, 6, 7]. Direct pathways encompass incidents directly link to weather or climate, such as frequency and intensity of extreme weather events like storm, flood, drought, or heat wave [8–10]. Indirect effects arise from complex ecosystems mediated by the consequences of environmental shifts including changes in the prevalence, location and severity of infectious diseases, air quality, food, and waterborne diseases [3, 8]. Furthermore, social human institution-mediated impacts such as climate-driven conflict, extreme weather events, economic dislocation, or environmental degradation also have a role [3, 8, 10].

To address this, countries and cities must develop policies or strategies aimed at reducing vulnerability to the future impacts of climate change [15]. The extent to which known health effects are influenced depends on the degree of implementing proactive, timely, and effective adaptation strategies [16]. Worldwide, there is growing interest in adaptation within health disciplines [17], yet substantial adaptation gaps exist and may widen as climate change increases [16]. Australia faces a unique risk to the health of the community, as the country is highly vulnerable to climate and weather extremes [17–19]. Climate change is adversely affecting the health of Australians and the country consistently finds itself unprepared for its consequences [4]. Australia must make adequate preparations to address the numerous health impacts expected to emerge from the changing environment. Managing some of these health risks will require preventive adaptation approaches that demand extensive coordination and collaboration amongst diverse stakeholders [2]. Furthermore, operationalising climate change adaptation (CCA) presents challenges [20] and will likely require innovative approaches to comprehend the complexity [21]. Multiple opportunities exist to build climate health resilience [22]; however, knowledge gaps exist in how Australia can prepare for climate hazards including weather-related disasters.

Consequently, Australia lags behind other countries in the implementation of public health aspects of CCA. Therefore, this paper aims to provide insights into approaches for addressing CCA and hence, set two objectives. Firstly, an integrative review to capture a comprehensive understanding of tools, frameworks, guidance material, or methods relevant to CCA and appropriate for preventative public health measures. Secondly, to explore key themes that can assist are discussed to bridge knowledge-action gaps in the context of Australian CCA for public health. To the authors’ knowledge, this review is the first of its kind to approach this topic from the perspective of preventative public health for climate change within the Australian context.

## Materials and Methods

An integrative review was conducted as described in Whittemore and Knafl [24] which includes problem identification, literature search, data evaluation, data analysis, and data presentation. This type of review is particularly suited to capturing information on a broad and diverse range of sources both empirical, methodological, and theoretical [25]. To guide data extraction, we employed the (i) Populations; (ii) Exposures; and (iii) Outcomes (PEO) framework, which is well suited for public health reviews as it aims to enhance the understanding of relationships between variables [26]. The Boolean operators ‘OR’ was used to combine search terms within each concept and ‘AND’ was used to combine all three concepts. We used a combination of Medical Subject Headings (MeSH) terms and keywords related to public health, climate change, and adaptation (refer to Attachment 1).

**Fig. 1:**
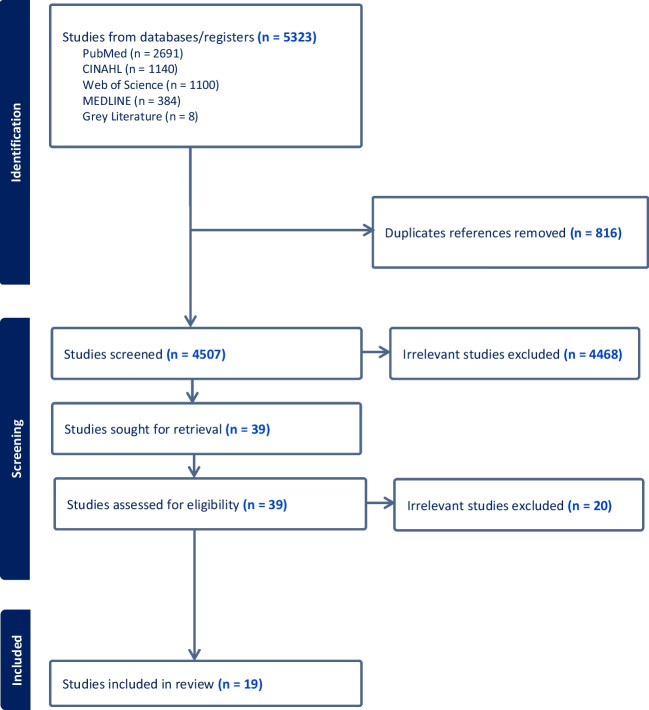
PRISMA [28] flowchart detailing the articles included in the integrative review at abstract, full text, and extraction phases

A primary search was conducted and finalised in January 2023 utilising international electronic databases (MEDLINE, PubMed, CINAHL, and Web of Science) with a date range between 1st January 2012 and 31st December 2022. Articles were restricted to the inclusion and exclusion criteria (refer to Table 1). The primary search yielded 5315 articles, which were then imported into Covidence systematic review software, and 816 duplicates removed. Subsequently, we conducted a secondary search in Google Scholar to identify additional literature for relevant academic publications or grey literature. Google Scholar sensitivity can pose problems by generating a low duplication rate for systematic searching [27]. To avoid the significant resource wastage manually screening thousands of articles [27], we chose to limit the Google Scholar review to the first 200 articles. Additionally, to maximise coverage of relevant examples, we conducted searches on the websites of reputable organizations such as IPCC, World Health Organization (WHO), and the United Nations Office on Disaster Risk Reduction (UNDRR). This secondary search was chosen because recent comprehensive international discussions may not have filtered into journal articles when the review was conducted. The secondary search yielded 8 articles for screening, resulting in 4507 articles to be screened. Next, the retrieved articles underwent a title and abstract review by the main author, for potential relevance meeting the inclusion criteria. This resulted in a further 4468 irrelevant articles being removed, leaving 39 articles for a full-text review. Then, a full-text review was undertaken by two independent reviewers (T.W. and R.P.) to determine if the article was meeting the inclusion criteria and categorised either ‘include’ or ‘exclude’. In cases of disagreements between the two reviewers, these were resolved and articles were evaluated by an independent third member (E.C.) of the research team. From the 39 full-text reviews, 19 papers were identified as meeting the inclusion criteria for this review (refer to Attachment 2). The following PRISMA diagram (refer to Fig. 1) summarises the screening process undertaken for this review [28].

### Selection Process 

Table 1

**Table 1:** Inclusion/exclusion criteria for review

Inclusion	Exclusion
• Population — public health OR environmental health practices	• Population — NOT discussing public health OR environmental health practices
• Exposure — climate change OR natural hazard OR extreme weather OR disaster OR vulnerable OR vulnerability	• Exposure — NOT discussing climate change OR natural hazard OR extreme weather OR disaster OR vulnerable OR vulnerability
• Outcome — climate change adaptation OR adaptive capacity OR resilience OR disaster risk reduction	• Outcome — NOT discussing climate change adaptation OR adaptive capacity OR resilience OR disaster risk reduction
Written in English	Non-English
Full text	Not full text, only abstract
Date range 1st January 2012 to 31st December 2022	Published outside the specified date range
Preventive public health focused	Articles focused on clinical or nursing or psychology fields or centred on immediate disaster response and/or recovery
Focus on broad human health impacts	Focused on primary health effect such as infectious disease
Operationalised approaches, frameworks, models, tools, guidance material	Not containing operationalised material such as background information
Primary search peer reviewed only	Primary search non-peer reviewed

### Data Analysis

The final articles were appraised for quality and general adequacy using the Joanna Briggs Institute (JBI) Critical Appraisal tools [29]. For each article, the design and methodology were assessed for the most appropriate JBI appraisal checklist and all articles were assigned to the Text and Opinions Checklist. This checklist tool includes six domains: source, expertise, relevant population, logic, reference to the literature, and incongruence with the literature [29]. In the critical appraisal procedure, the main author evaluated the 19 full‐text articles for their quality. An assessment of the quality of the Text and Opinions Checklist was undertaken using each item and was given a criterion (yes, no, unclear, and not applicable). The decision about the scoring system and the cut‐off for the inclusion of a publication were made in advance by the full-text reviewers (T.W. and R.P.) before critical appraisal commenced. It was decided that the study had to meet more than 50% of the assessment criteria be considered for inclusion. Overall, the quality of the articles was considered good (refer to Attachment 2), if the article clearly identified five out of the six criteria. No studies were excluded due to low quality.

## Results

### Description of Studies Selected

**Fig. 2:**
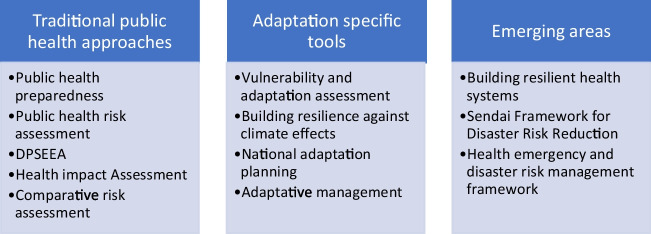
Three main categories and results of the identified preventive public health approaches for climate change adaptation

In the scientific literature, ten articles identified were mainly contextualised globally [7, 15, 21, 30–36] while three others were from the USA [37–39]. The methodologies used to address CCA were eight report/commentary [7, 30, 32, 34–36, 38, 39] while five others were reviews [15, 21, 31, 33, 37]. Articles found through the grey literature search have different characteristics than the scientific literature. WHO was the main organisation providing guidance material on health adaptation and health systems (4 of 6 articles) [3, 22, 40, 41]. United Nations of Disaster Risk Reduction [42] and WHO [43] provided a report each on the Health Emergency and Disaster Risk Management Framework (see attachment 2). The preventive public health approaches to CCA identified in this review were grouped into three main categories: traditional public health approaches, adaptation specific, and emerging areas (refer to Fig. 2).

### Traditional Public Health Approaches

Traditional public health approaches are established and well-known methods used in the public health practice to assist in minimising adverse health impacts.

#### Public Health Preparedness

The prevention principle plays a central role for the public health preparedness to minimise health impacts, especially in the face of scientific uncertainty [44]. There are clear similarities between CCA aimed at reducing health impacts and the conventional public health preparedness practices of primary, secondary, and tertiary prevention [7, 44, 45]. Primary prevention aims to intervene before the onset of disease or injury in reducing the exposure to the hazard [7, 35, 45]. Secondary prevention has the intention to detect health-related changes, then take specific action early to avoid adverse health consequences once disease or illness has commenced [35, 44, 45]. Tertiary prevention aims to have an effective response once a disease or illness has been diagnosed and is therefore the most reactive strategy [35, 44, 45].

However, some authors suggest adding a fourth prevention principle, primordial (or pre-primary) as the first line of prevention [35, 46]. Primordial prevention involves preventing health exposures by modifying the broad health determinants that can amplify the risk of disease [35, 46]. This approach addresses systemic factors of preventing individual exposure to risk factors [35, 46]. For instance, this prevention strategy can be used to guide development decisions for settlements in hazardous areas such as floodplains also aligns with the disaster risk reduction (DRR) approach [35]. These four prevention principles are interconnected and contribute to development climate change responses. For example, this can involve modifying underlying health determinants, reducing risk factors before exposure, preventing adverse health outcomes from exposure, and reducing the long term of the health impact.

#### Public Health Risk Assessment

Health practitioners often use risk assessments to determine whether a proposal or activity will adversely affect the health of the population. These assessments account for all substances that people are exposed to and how they interact [47]. Typically, risk assessment adheres to core principles involving: issue identification, hazard assessment, dose–response relationship, exposure assessment, and risk management [47]. The scope of public health risk assessment can vary across several health impacts and there are several different approaches to undertaking the assessments. Toxicological risk assessments involves estimating the degree and probability of adverse health effects to humans that are exposed to various types of hazards [48]. Epidemiological risk assessments focus on the proximate risk factors and adopt a reductionist approach to managing the risk [49]. These conventional public health risk assessments have successfully controlled single contaminant or exposure pathway while explaining the health outcome [23, 49]. However, traditional public health risk assessment, while valuable, the narrow framing has limitations applying to complex and cascading climate change health risks [49]. The assessment also lacks guidance on prioritising interventions or evaluating their effectiveness across a range of climate-sensitive health outcomes [49].

#### DPSEEA

**Fig. 3:**
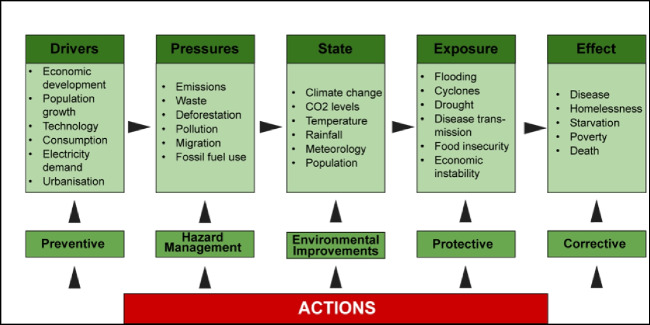
DPSEEA sequence of events contributing to an environmental health problem interpreted for the disaster context (adapted from Waheed, Khan & Veitch 2009) [53]

The Driving Force, Pressure, State, Exposure, Effect, Action (DPSEEA) framework of the WHO has been widely applied to highlight the link between environmental degradation and human health [50]. This framework is used to systematically describe and examine a logical conceptual sequence of events contributing to an environmental health problem [51]. For instance, driving forces indirectly place pressure on the environment, which alter its state to produce negative influences on health [52] (refer to Fig. 3). In addition, the framework was designed to support decision-making on actions to reduce health impacts from environmental or related behavioural conditions [52]. The DPSEEA framework also aligns in some ways with the four prevention principles in the public health preparedness discussed earlier.

The main strength of this framework lies in its clear recognition to the various intervention points that can be applied along the environmental-health causal chain [54]. This framework could assist public health actors to identify the range of potential health effects resulting from changes that would require further consideration when undertaking vulnerability assessments later [15, 52]. The DPSEEA framework has already been adopted in proposals for monitoring health impacts of climate change [55]. Additionally, the framework is considered particularly suitable for developing environmental health indictors for assessing and monitoring human health vulnerability of climate change [51]. In Australia, the New South Wales government has recently incorporated this framework in the Climate Change Policy Framework, to guide appropriate public health adaptation to climate change [50].

The challenge of using this framework is the difficulty in representing the complex and diverse causal web that links climatic, environmental, and social determinants affecting human health [56]. For example, climate change involves multiple and complex pathways as well as interactions between climatic and non-climatic risk factors influencing human health [56]. The DPSEEA framework can simplify reality by focusing on linear pathways between environment and health, potentially leading to the oversight of the actual complexity of these relationships and the pursuit of simplistic solutions to multifaceted problems [54]. To make the framework more practical, it has been suggested the process should involve adapting and modifying to include intermediate ecological indicators and relevant non-climatic confounding health risk factors [56].

#### Health Impact Assessment

Health Impact Assessment (HIA) is a process that utilises a combination of procedures, methods, and tools to assess the potential health impacts of a policy, programme, or project [57]. The recommendations from HIA allow decision-makers and stakeholders to maximise positive health effects while also minimising any negative health effects [58, 59]. This assessment is flexible with a multi-disciplinary and collaborative approach to include the input and expertise of a range of public health and other stakeholders as a means for building of healthy public policy [59, 60]. HIA has become an additional assessment tool useful in anticipating how climate change can affect population health from policy, projects, or programmes being used across a range of sectors [52, 59]. Usually, the assessment can potentially identify and communicate important health impacts before their implementation with a preventive health perspective [15, 52]. The Western Australia Department of Health demonstrated the applicability of HIA to consider the implications of climate change on their population and to develop a range of adaptation strategies [61].

The assessment will require intensive data from various networks to deliver robust health assessment tools [15]. HIA process requires access to valid data and often involves a complex process, requiring thorough systematic methods in predicting these health impacts [59]. The broad stakeholder engagement required for HIA is a very time-consuming exercise [62]. This timing issue could be problematic in the highly polarised political environment where governments could change every few years to cause a significant reversal of policy and with an emphasis on cost cutting [63]. Furthermore, it can be challenging to decide what adaptation strategies will most effectively reduce vulnerability and where those actual measures be targeted [64].

Australia was once considered an international leader in developing HIA, but the progress since has been inconsistent around the country [65]. HIA remains poorly integrated into policy development and decision-making in Australia, as there is limited legislative support in considering the health impacts [66]. Systemic capacity building across three key areas is required for HIA to improve as tool for CCA [65]. These three areas are as follows: (1) developing strong political and governmental support; (2) using robust instruments during the process; and (3) building workforce capacity to undertake as well as evaluating HIA [65].

#### Comparative Risk Assessment

Comparative Risk Assessment (CRA) is a tool to inform public health policy by using a comprehensive approach to risk factor quantification by examining evidence on risks and their outcome associations [67]. This assessment is defined by WHO as ‘the systematic evaluation of the changes in population health which result from modifying the population distribution of exposure to a risk factor or a group of risk factors’ [67]. The aim is to estimate the burden of disease that is attributed to a risk factor and the burden that is preventable by possible reductions in the risk factor [67]. National policymakers could use CRA quantitative disease estimates to determine the most appropriate strategies that climate change may cause to specific populations in adaptation planning [68]. Many countries have used Global Burden of Disease information to guide health policy decisions and to determine prevention measures to improve population health [69]. However, there appears to be scant evidence of CRA use for National CCA relating to health outcomes. There are limitations of these assessments, for example quantitative estimates are highly uncertain and very data intensive [68, 70]. The influence of non-climatic risk factors, such as social health determinants, is not well considered in the assessment process could lead to an underestimation of health impacts [68, 70].

### Adaptation-Specific Tools

Adaptation-specific tools are specialised instruments that are intended to facilitate the adaptation process to assist in minimising adverse health impacts.

#### Vulnerability and Adaptation Assessment

Undertaking Vulnerability and Adaptation Assessments (V&As) is crucial to evidence-based information when implementing a risk management approach to effectively address climate change health impacts [33, 41]. The assessments are built on the core principles of risk assessment, i.e. issue identification, hazard assessment, dose–response relationship, exposure assessment, and risk management, as discussed earlier, in considering the threats posed by climate variability and change [41]. Furthermore, the assessment enables decision-makers to understand the magnitude of potential impacts on health from climate change as well as evaluating the effectiveness of implemented strategies [3, 33, 71]. The assessment recommendations aim to strengthen health systems and build resilience of people vulnerable to the impacts of climate change health risks [41]. The V&A process is iterative in the preparation and response to ongoing changes, rather than being a standalone activity [41]. Undertaking the assessment provides an opportunity for the collaboration to occur between key health authorities at all levels and non-health-related stakeholders in delivering recommendations with a joint understanding [52].

Undertaking local or regional assessments is beneficial as they can identify locally specific health risks together with appropriate local response which could be overlooked under a national context [33]. This will provide evidence-based information for the understanding for a local community health risk and to navigate the challenges posed and respond effectively [33, 49]. To provide improved outcomes from the assessment of the health effects, systemic, synergistic, cascading risks must be included as part of V&As for a holistic understanding of the potential impacts [33]. To assist national efforts to assess the climate change impacts on health, WHO framework and guidance for public health adaptation for conducting V&As have been developed [41]. However, guidance material and robust indicators to track progress in building resilience for subnational levels are lacking [72, 73].

Several challenges exist in undertaking these V&A assessments or in meeting their full potential. Deciding suitable timescales is a complex applicable to decision-makers and distinguishing clear short-term and longer-term adaptation options [33, 52]. A review of V&As undertaken by WHO revealed that the qualitative or quantitative projections to include future risk projections of health impacts are lacking [33]. Often, the assessments are narrowly focused on building an evidence-base on specific disease burdens and require more comprehensive analysis of broader components of the health system [33]. In some assessments, vulnerability aspects are often missed, and epidemiological data and climate scenarios were also inadequate [33]. The translating of findings into implementable practice is problematic due to funding allocation and limited capacity or is time consuming [33, 52]. It has been argued that V&As only have some influence over policy prioritisation in some countries [17]. Lastly, data accessibility of different types such as climate parameters, health conditions, socioeconomic profiles, or institutional capacity causes limitations in predicting health outcomes [15, 21].

#### Building Resilience Against Climate Effects

Ten essential services (refer to Table 2) provide public health agencies in the USA with a framework that outlines their basic responsibilities and are the foundation for the Nation’s public health strategy [74]. This simple framework is flexible enough to be used by many state and local health agencies and can be applied to various contexts in the USA [75] as well as underpinning their national Climate and Health Program (CHP) [37]. As part of CHP, new tools were developed including an assessment framework in supporting state and local government efforts towards climate health adaptation [37, 76]. The Building Resilience Against Climate Effects (BRACE) framework has been developed based on lessons learnt from global V&As on how to improve their effectiveness in the context of health systems [49, 76]. In particular, adaptive management combines a learning-focused, iterative approach to allow for an adjustment step to reflect the dynamic uncertainty within complex systems [21]. The five-step process involves identifying vulnerabilities, projecting disease burdens, assessing the interventions to lessen the health risks, developing a health adaptation plan, and evaluating implemented decisions [39, 49].

**Table 2:** Ten essential public health services 2020 revised version [74]

1. Assess and monitor population health status, factors that influence health, and community needs and assets
2. Investigate, diagnose, and address health problems and hazards affecting the population
3. Communicate effectively to inform and educate people about health, factors that influence it, and how to improve it
4. Strengthen, support, and mobilise communities and partnerships to improve health
5. Create, champion, and implement policies, plans, and laws that impact health
6. Utilise legal and regulatory actions designed to improve and protect the public’s health
7. Assure an effective system that enables equitable access to the individual services and care needed to be healthy
8. Build and support a diverse and skilled public health workforce
9. Improve and innovate public health functions through ongoing evaluation, research, and continuous quality improvement
10. Build and maintain a strong organisational infrastructure for public health

A modified version may increase the value of the BRACE framework, by combining an ‘adaptation pathways approach’ [77]. This approach could be included to consider longer timescale uncertainties for a more robust decision-making and overcome some of the barriers to climate change [7]. Including this pathways approach provides a suite of options from the start, which leads to an end point of where the system needs to be [7]. This enables in short-term decisions to be flexible, while the subsequent and appropriate actions can be taken over time to reach the goal [7]. The BRACE framework has been considered very helpful and powerful for public health system adaptation, particularly at local level [7, 21]. However, the framework is targeted to the settings of USA and would need to be significantly modified to reflect the local health system for it to be useful in the Australian context.

#### National Adaption Plan

The United Nations Framework Convention on Climate Change (UNFCCC) encourages countries to prepare a National Adaptation Plan (NAP) for medium- and long-term CCA and implement strategies to address those needs [78]. The process assists countries to understand climate change risks, vulnerability, and adaptation options, as well as prioritising and implementing strategies [79]. An Australian NAP could be useful as being the main driver in implementing adaptation strategies in health [31] and also contribute to strengthening of the entire health system [6, 40].

**Fig. 4:**
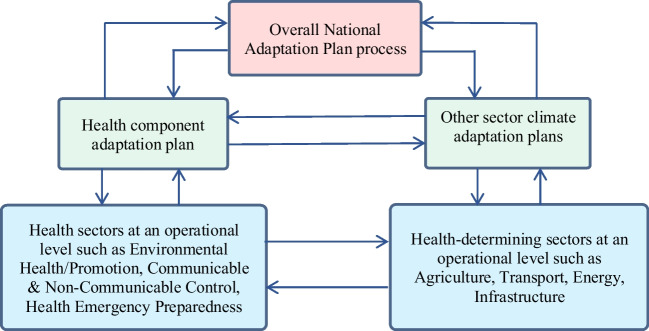
Summary of the health sector involvement in the National Adaptation Plan process. (Adapted from Quality criteria for health national adaptation plans 2021) [3]

To adequately and effectively maintain the health of the community for future climate change risk, it is important that the health component is appropriately considered by developing the Health-National Adaptation Plan (H-NAP) [3]. Undertaking comprehensive V&As is critical in providing evidence-based H-NAP and is essential for providing information for policymakers [3]. The health component ensures that the health risks of climate change are prioritised to reduce vulnerabilities and build needed capacity and resilience [34]. If health is not fully represented during the process, this may overlook essential strategies in protecting population health or resulting in the implementation of programmes from other sectors which may unintentionally cause adverse health impacts [3]. Therefore, a participatory approach would be necessary for a more effective H-NAP in considering contributions from many stakeholders at different levels, across sectors and the local community [3]. This can assist determining the cross-sectoral institutional arrangements and recognise gaps or needs requiring additional measures for the coordination and improving synergy of other health-determining sectors [3, 80] (refer to Fig. 4).

Australia does not have a NAP but instead has the National Climate Resilience and Adaptation Strategy 2021–2025 [5]. The health component in this strategy is largely inadequate and health is mentioned sparingly [81]. For instance, a comprehensive V&A has not been undertaken which resulted in the plan not having strategies designed to address the adverse health risks or a detailed goal with a strong focus on health action. However, in the absence of meaningful national policy, state and territory governments are leading the way in developing their own health and climate change adaptation planning [4, 5]. For example, the state of Queensland developed a specific regional scale climate adaptation plan with the health sector [82].

#### Adaptive Management

One challenge of public health is in developing practical responses while encountering complexity and uncertainty as well as applying the ever-changing factors such as advances in new knowledge [21, 37]. These are key barriers for public health adaptation indicating that linear approaches for decision-making are likely inadequate and a more flexible approach is required [7]. Adaptive management (also known as iterative risk management) enables the assessment of multiple decisions, and this may have a significant possibility for public health adaptation to anticipate, plan for, and respond to the health risks in building resilience [21, 83]. This type of iterative approach acknowledges that complex adaptive systems are rarely fully understood and making it suitable for decision-making in circumstances involving uncertainty [21, 37, 52, 83]. In addition, the uncertainty settings allow for active learning to assist in developing multiple viewpoints which can lessen the uncertainty over time and need to adjust the system according to changes [21]. The process continues to evolve by combining collective loop learning framework to enable the evaluation of implemented decisions, allowing to incorporate new knowledge and new opportunities to be considered [21, 73]. Adaptive management has been used in other sectors, such as natural resources systems management, and is expected to be also useful in CCA for public health [21, 83].

### Emerging Areas

Emerging areas are novel approaches that can assist in minimising adverse health impacts but are not yet found in the mainstream public health practices.

#### Building Resilient Health Systems

Climate change will impact upon climate-sensitive health outcomes, but also requires the delivery of appropriate public health programmes in a climate-resilient health system [8, 22]. A key strategy of adaptation is health system resilience to the effects of climate, as described in the WHO’s Operational Framework for Building Climate Resilient Health Systems [22]. The process involves understanding the strengths and weaknesses of existing health systems, as well as the ability to modify programmes in response to changes or impacts [8, 22]. The framework comprises of 10 components with six interconnected building blocks of health systems [22] (refer to Table 3). These components should also feature in developing comprehensive H-NAP with resilience which is the goal of the framework [22]. Building a climate-resilient health system to improve health of the population involves the capacity of anticipating, responding to, cope with, recover from, and adapting to climate-related shocks and stresses [22]. Appling the framework in full has the potential to accomplish transformational adaptation which fundamentally changes the systems and addresses root causes of vulnerability [16]. However, within the complex world, it is difficult to determine exactly what a health system is and the boundaries of what it consists of and where it starts and ends [84, 85]. Health system resilience is also ambiguous and is highly confused as resilience means different things for different people [86].

**Table 3:**
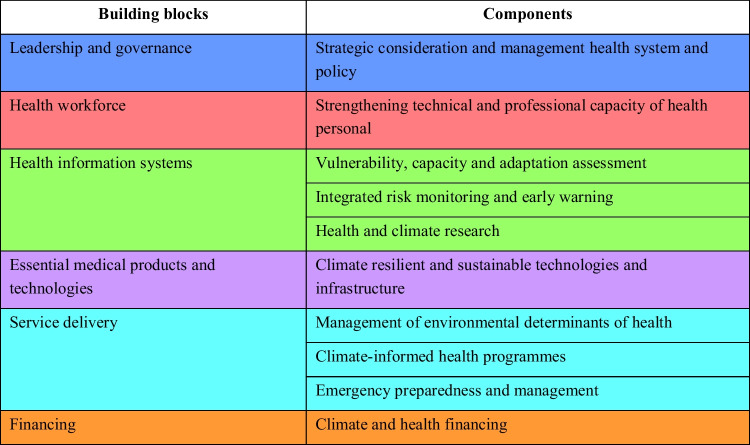
Ten components and the building blocks to strengthen the health system. (Adapted from Operational framework for building climate resilient health systems 2015) [22]

#### Sendai Framework for Disaster Risk Reduction 2015–2030

Climate change is a driver for increasing frequency and intensity for particularly weather-related hazards and has identified the need for an improved understanding of the interactions between climate change and disasters [30, 31, 34]. The Sendai Framework signifies the push in global policy coherence by having explicit reference to health, economic development, and climate change for a more equitable, resilient, and sustainable future [30, 36, 49, 87, 88]. Expanding the disaster definition has emphasised an all-hazards approach to include health-specific hazards such as biological hazards and epidemics [30]. From a preventive health perspective, consequences of disasters are most effectively minimised by reducing the disaster risks, reducing hazard exposure, minimising vulnerabilities, and/or increasing capacities [43]. In contrast to more traditional approaches to disaster management, this framework focuses on systematic analysis to manage health and disaster risks [42].

There are commonalities and considerable overlaps of different health impact pathways of climate-sensitive disasters and climate change [32, 36]. Therefore, strategies to reduce the climate-sensitive disaster and climate change health risks should also have a similar outcome [32]. Integrating DRR and CCA strategies has been suggested to be necessary for a robust approach in strengthening health system, building resilience while reducing risk as well as vulnerability [31, 34, 36]. The public health sector has an important and vital role in establishing and sustaining the connection between DRR and CAA [31, 48]. However, ongoing challenges to integrating CCA and DRR exist such as lack of capacity amongst key stakeholders and a lack of coordination and collaboration across scales of government and funding [89]. Additionally, it is also hampered by the fact that health resilience is often not part of the discussion in building these links at the national policy level nor with various sectors [31]. This demonstrates that there is limited understanding of the importance of building the links in health and the in-depth knowledge required is lacking [31].

In Australia, the National Disaster Risk Reduction Framework provides a strategic approach for stakeholders to reduce climate and disaster risks in all sectors of the society [90]. However, disaster management in Australia is complex and suffers from a fragmented policy environment, with inefficiencies and lack of resources or capacity to implement an integrated approach [91, 92]. The mainstreaming process of a key stakeholder of local government participation in DRR and CCA remains low [91]. This local level participation in health is particularly important as this is where services are mainly provided to the community and the sector has the appropriate knowledge and information of what is needed [38].

#### Health Emergency and Disaster Risk Management Framework

There has been a lack of coherency and consistency to embed health in disaster risk in the Sendai Framework, from fragmented approaches and the continual over-emphasis on reacting to hazardous events [42, 43, 93]. As a result, WHO developed the Health Emergency and Disaster Risk Management Framework (Health-EDRM) to assist in facilitating health resilience, to strengthen health system, and to consolidate existing contemporary practices [43, 93]. Additionally, mainstreaming health as part of the framework will aid in supporting policies and actions for the international risk-resilient and sustainable development agendas which can also build convergence and synergy [42, 43, 93]. Health-EDRM also aims to embed within existing health systems to leverage greater emphasis on risk prevention and building health resilience at local to national levels as well as preparedness, response, and recovery [93].

## Discussion

The review demonstrated that climate change risks have the potential for adverse consequences for both human and ecological systems [94]. These impacts are a result of dynamic interactions between climate-related hazards, exposure, and vulnerability which can vary over time or with socio-economic change [94]. Developing effective adaptation strategies for health will require a systematic understanding of which individuals or populations are vulnerable, and how different pathways contribute to their vulnerability [52, 95]. This view supports vulnerability as a cause of increasingly interconnected, cumulative, and cascading nature of risk with a potential impact on health, social, economic, and other systems [96]. Therefore, adopting complex systems thinking is required in considering cascading risk factors and their interconnections as well as accounting for several possible known causes [33, 97]. This dynamic nature of risks within complex systems must be considered while undertaking health risk assessment frameworks for CCA to address the root causes of vulnerability and the building health resilience [37, 98–100]. Yet, in Australia, conventional linear approaches to risk management largely persist, which will be inadequate to address the climatic health risks.

The challenges posed by the nature of systemic risk will require multi-system systems perspectives while applying system thinking to be effective in managing risk [49, 97, 99]. This has been increasing recognised that integrative approaches that embrace a broad understanding of the complexities across environmental, social, and human systems are required to effectively managing the climate health risks [2, 49, 83, 99]. The concept of a socio-ecological system approach focuses on the notion that social and natural systems are not only closely related and fundamentally integrated as well [101]. Under climate change, health issues do not exist in isolation nor can be separated from socio-ecological systems [102]. This supports the value of system-base understanding for climate risk management and response options targeted towards maintaining population health [8, 16]. However, research in systems-based understanding of the complex interaction of climate-related hazards and in developing effective responses to reduce or prevent the risk of adverse health outcomes is lacking [3, 49, 83]. System thinking for public health practice in Australia has thus far been challenging because of the preference in pursuing medical models of disease causation [49]. This also resonates with the limitations of typical reductionist approaches to epidemiology, which focus more on the individual direct causes with little attention to indirect health determinants affecting the whole of the population [49]. These direct approaches will be insufficient to understand and manage the health risks of climate change within complex adaptive systems.

**Fig. 5:**
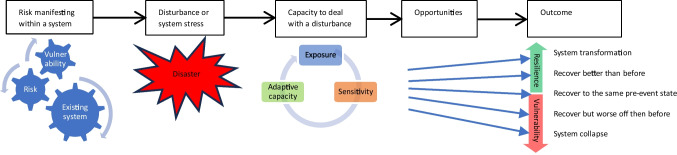
Possible system pathways after a disturbance. (Adapted from Operational Framework for Building Climate Resilient Health Systems 2015) [22]

As climate change continues to increase over the years, this will also likely to further exacerbate the dangerous risks to human health [16]. Consequently, there will a greater emphasis on transformational change to build resilience of health and other systems [16]. Transformation is achieved when adaptation programmes alter the fundamental approaches to be better prepared for cascading health risks by addressing the root causes of vulnerability [87]. This was highlighted recently in the New South Wales town of Lismore, where a section of the community was already well known for their existing vulnerability to flooding. In 2022, Lismore suffered back-to-back major flooding events, a month apart, which resulted in deepening and reinforcing the existing state of vulnerability. As a result, Australia relies on the returning to, and supporting, the status quo in saving lives and assets for disaster situations [98]. This does not allow for system transformation and existing vulnerabilities are entrenched in community to become more vulnerable (refer to Fig. 5). Yet, priority four in the Sendi Framework suggests disaster recovery phase is a critical opportunity for ‘Building Back Better’ and to transform in becoming more resilient to future disasters [103].

Over the years, disaster management has shifted in focus from primarily being response and recovery, to a more holistic approach of incorporating prevention, preparedness, response, and recovery [88]. This focus has continued to evolve, with the Sendai Framework focusing on reducing the risk of a natural event becoming a disaster by improving resilience and eliminating hazards. Historical data relating to previous catastrophic disasters have shown that socio-economic factors can be significant in determining the likelihood of disaster potentially impacting on health [104]. The legacy of past decisions, political structures, poor design and construction of buildings, and other factors make some communities more susceptible to adverse health outcome of disasters [41, 98]. The contemporary position is that disasters are not naturally occurring but are instead more appropriately considered to be socially constructed within the community contributed from human activities [105, 106]. The consideration of the social and environmental determinants of health in DRR planning can assist the capacities of decision-makers to essentially manage and reduce future risk while improving current health status [107, 108]. Developing a holistic understanding by considering determinants of health in DRR and planning for those who are most vulnerable to these impacts will assist in the planning and targeting of interventions [31, 107, 108]. Consequently, public health needs to employ a proactive approach, with risk reduction strategies addressing the upstream root causes that create social vulnerabilities [108]. To date, these prevention approaches have largely been neglected in the Australian context and instead public health is primary viewed as a response function [109]. For instance, 97% of all Australia disaster funding is heavy targeted towards relief and recovery [110]. This is significantly missing prevention opportunities in apply risk reduction strategies to minimise natural hazards becoming disasters [110].

Despite Australia’s vulnerability to health impacts of climate change, the political engagement on climate change and health at the national level has been historically poor [5, 18, 19]. In the past, the Commonwealth Government’s primary engagement was not even focused on their own population but mainly providing disaster response to nearby Pacific Island nations [111]. There is clear evidence suggesting that adequate investment in preventive public health can be beneficial to the health system and be more cost effective to tertiary hospital care [112]. However, Australian total healthcare expenditure relating to public health is only 1.6% [113] and is considerably lower compared to other similar OECD countries [114]. This highlights investment in preventive public health is significantly inadequate and continues placing great pressure on Australia’s healthcare system [115]. Despite the potential for significant returns from investments into preventive health, there is substantial disparity in funding compared to clinical services in Australia [116]. Furthermore, capacity constraints to address CCA are also evident as a result of years of inadequate funding in public health and disease prevention [117].

Policymakers must begin preparing now for this future to integrate climate and disaster health risks to understand the compounding and cascading effects in moving away from business-as-usual approach [110]. However, the Australian Government’s reductionists system is not well suited to address complex problems like climate change nor is suitable for the required integrated responses across all levels of government [63, 92]. For instance, the responsibility for managing complex issues encourages working in silos and duplication of activity as well as causing jurisdictional disputes or lack of trust amongst government levels [5, 63]. The implementation gap of integrating CCA and DRR will continue to widen unless there is significant governance reform and more resources for better engagement with vulnerable populations [92]. Climate and Health Alliance is Australia’s peak body on climate and health has developed and has since updated an evidence-based framework on climate, health, and well-being for Australia [121]. This framework could potentially provide the necessary leadership for the Commonwealth Government as a foundation for a comprehensive national strategy on climate and health [121]. However, the framework has still not been adopted by Commonwealth Government which requires the political will and resourcing to support the implementation [122]. For the government to address CCA, will require holistic, integrated, and cross-scale responses at all government levels in moving beyond the current entrenched silos [63, 121]. Public health also needs more integration into DRR and CCA policymaking processes and build partnerships and to facilitate cross-sectoral collaborations [123].

The Health in All Policies (HiAP) framework promoted by WHO is for public policies across sectors to collaborate and systematically account for the health implications of decisions in policy-making [124]. This enables other sectors to consider the impact their own work has on health, while breaking down silos in building partnerships to promote health, equity, and sustainability as well as increase government efficiency [125]. Several health risks are affected or even determined by policy decisions in sectors outside the health sector [60]. For example, policies applied to energy generation and transport planning have multiple health co-benefits to consider [60, 126]. The framework is suited to a variety of complex and often inextricably linked problems to enable root cause mapping in identifying key factors contributing to community health issues [127]. The HiAP approach has increasingly been used globally to consider the multi-sectoral aspect of health impacts from climate risks or hazards in all policies and across all sectors [124, 126, 128]. However, this will require committed and visionary political leaders to foster a new approach in how government functions to reach its full potential. The state of South Australia is considered an international leader on using this approach [124, 127] but in general, Australia has lagged compared to the rest of the world in adopting HiAP. This may increase the Commonwealth Government engagement in linking climate change and health which is critical for achieving meaningful and coordinated action from all levels of government [5].

## Limitations

This integrative review does have some limitations, both in the methodology and the application of the methodology. While a rigorous methodological approach was used, some relevant papers may have been overlooked and therefore not included in the analysis. The keywords and search strings used may have missed articles because some articles may have applied different keywords. Additionally, the primary review only included four databases and searching further databases could have uncovered more appropriate articles. Lastly, only articles written in the English were included. Low- and middle-income countries are more disproportionately affected from climate change which may have resulted in publishing in their national language. Despite these limitations, the authors believe that this review will provide a comprehensive starting point for public health in understanding and the urgency of CCA implementation.

## Conclusion

Conventional linear approaches to risk management once successful in addressing problems are ill-suited in solving today’s complex health issues. System thinking is required to move from understanding disease causation from a medical perspective, to instead recognising the complex risk interactions of health determinants. Additionally, taking a complex adaptive thinking approach will also be required that investigates problems holistically for solutions. This review explained the necessary preventive public health tools available for Australia to undertake risk reduction strategies to address the upstream root causes of vulnerability. There are also approaches which, when integrated, are key to sustainable development and effectively managing health impacts of climate change and disasters. To date, Australia’s progress in health adaptation has been poor and will require ‘playing catch-up’ compared to countries with more experience in climate change planning. This will require political engagement, governance reform, and more funding for preventative public health to urgently build community health resilience to the changing climate. Until this occurs, Australia will continue to be caught underprepared from the health risks of climate change and climate-sensitive disasters.

### Supplementary Information

Below is the link to the electronic supplementary material.

Supplementary file1 (DOCX 25.7 KB)

## Data Availability

All data supporting the preventive public health findings of this manuscript are available within the paper and its Supplementary Information (refer to Attachment 2).

## References

[1] International Panel on Climate Change. Summary for policymakers. In: H Lee & J Romero, editors. Climate change 2023: synthesis report. a report of the IPCC. contribution of working groups I, II and III to the sixth assessment report. Geneva: IPCC; 2023. 10.59327/IPCC/AR6-9789291691647.001

[2] Machalaba C, Romanelli C, Stoett P, Baum S, Bouley T, Daszak P, Karesh W (2015). Climate change and health: transcending silos to find solutions. Ann of Glob Health.

[3] World Health Organization (2021). Quality criteria for health national adaptation plans.

[4] Beggs P, Zhang Y, McGushin A, Trueck S, Linnenluecke M, Bambrick H, Capon A, Vardoulakis S, Green D, Malik A, Jay O, Heenan M, Hanigan I, Friel S, Stevenson M, Johnston F, McMichael C, Charlson F, Woodward A, Romanello M (2022). The 2022 report of the MJA–Lancet Countdown on health and climate change: Australia unprepared and paying the price. Med J of Australia.

[5] Heenan M, Rychetnik L, Howse E, Beggs P, Weeramanthri T, Armstrong F, Zhang Y (2023). Australia’s political engagement on health and climate change: the MJA–Lancet Countdown indicator and implications for the future. Med J of Australia.

[6] Romanello M, McGushin A, Di Napoli C, Drummond P, Hughes N, Jamart L, Kennard H, Lampard P, Solano Rodriguez B, Arnell N, Ayeb-Karlsson S, Belesova K, Cai W, Campbell-Lendrum D, Capstick S, Chambers J, Chu L, Ciampi L, Dalin C, Dasandi N, Dasgupta S, Davies M, Dominguez-Salas P, Dubrow R, Ebi K, Eckelman M, Ekins P, Escobar L, Georgeson L, Grace D, Graham H, Gunther S, Hartinger S, He K, Heaviside C, Hess J, Hsu S-C, Jankin S, Jimenez M, Kelman I, Kiesewetter G, Kinney P, Kjellstrom T, Kniveton D, Lee JK, Lemke B, Liu Y, Liu Z, Lott M, Lowe R, Martinez-Urtaza J, Maslin M, McAllister L, McMichael C, Mi Z, Milner J, Minor K, Mohajeri N, Moradi-Lakeh M, Morrissey K, Munzert S, Murray K, Neville T, Nilsson M, Obradovich N, Sewe M, Oreszczyn T, Otto M, Owfi F, Pearman O, Pencheon D, Rabbaniha M, Robinson E, Rocklöv J, Salas R, Semenza J, Sherman J, Shi L, Springmann M, Tabatabaei M, Taylor J, Trinanes J, Shumake-Guillemot J, Vu B, Wagner F, Wilkinson P, Winning M, Yglesias M, Zhang S, Gong P, Montgomery H, Costello A, Hamilton I (2021). The 2021 report of the Lancet Countdown on health and climate change: code red for a healthy future. Lancet.

[7] Wheeler N, Watts N (2018). Climate change: from science to practice. Current Environ Health Reports.

[8] Ebi K, Harris F, Sioen G, Wannous C, Anyamba A, Bi P, Boeckmann M, Bowen K, Cissé G, Dasgupta P, Dida G, Gasparatos A, Gatzweiler F, Javadi F, Kanbara S, Kone B, Maycock B, Morse A, Murakami T, Mustapha A, Pongsiri M, Suzán G, Watanabe C, Capon A (2020). Transdisciplinary research priorities for human and planetary health in the context of the 2030 Agenda for Sustainable Development. Int J Environ Res & Public Health.

[9] Schnitter R, Verret M, Berry P, Chung Tiam Fook T, Hales S, Lal A, Edwards S (2018). An assessment of climate change and health vulnerability and adaptation in dominica. Int J Environ Res Public Health.

[10] Watts N, Adger N, Agnolucci P, Blackstock J, Byass P, Cai W, Chaytor S, Colbourn T, Collins M, Cooper A, Cox P, Depledge J, Drummond P, Ekins P, Galaz V, Grace D, Graham H, Grubb M, Haines A, Hamilton I, Hunter A, Jiang X, Li M, Kelman I, Liang L, Lott M, Lowe R, Luo Y, Mace G, Maslin M, Nilsson M, Oreszczyn T, Pye S, Quinn T, Svensdotter M, Venevsky S, Warner K, Xu B, Yang J, Yin Y, Yu C, Zhang Q, Gong P, Montgomery H, Costello A (2015). Health and climate change: policy responses to protect public health. Lancet.

[11] United Nations Framework Convention on Climate Change. The Paris Agreement. n.d. https://unfccc.int/process-and-meetings/the-paris-agreement. Accessed 30 June 2023.

[12] International Panel on Climate Change. Summary for policymakers. In: V Masson-Delmotte, P Zhai, H Pörtner, D Roberts, J Skea, P Shukla, A Pirani, W Moufouma-Okia, C Péan, R Pidcock, S Connors, J Matthews, Y Chen, X Zhou, M Gomis, E Lonnoy, T Maycock, M Tignor & T Waterfield, editors. Global warming of 1.5°C. an IPCC special report on the impacts of global warming of 1.5°C above pre-industrial levels and related global greenhouse gas emission pathways, in the context of strengthening the global response to the threat of climate change, sustainable development, and efforts to eradicate poverty. Cambridge: Cambridge University Press; 2018. 10.1017/9781009157940.001

[13] United Nations Framework Convention on Climate Change. Climate plans remain insufficient: more ambitious action needed now. 2022. https://unfccc.int/news/climate-plans-remain-insufficient-more-ambitious-action-needed-now. Accessed 30 June 2023.

[14] Maibach E, Sarfaty M, Mitchell M, Gould R (2019). Limiting global warming to 1.5 to 2.0°C - a unique and necessary role for health professionals. PLoS Med.

[15] Delpla I, Diallo T, Keeling M, Bellefleur O (2021). Tools and methods to include health in climate change adaptation and mitigation strategies and policies: a scoping review. Int J Environ Res & Public Health.

[16] Cissé G, McLeman R, Adams H, Aldunce P, Bowen K, Campbell-Lendrum D, Clayton S, Ebi K, Hess J, Huang C, Liu Q, McGregor G, Semenza J & Tirado M. Health, well-being, and the changing structure of communities. In: H Pörtner, D Roberts, M Tignor, E Poloczanska, K Mintenbeck, A Alegría, M Craig, S Langsdorf, S Löschke, V Möller, A Okem & B Rama, editors. Climate change 2022: impacts, adaptation and vulnerability - contribution of working group II to the sixth assessment report of the IPCC. Cambridge: Cambridge University Press; 2022. 1041–1130. 10.1017/9781009325844.009

[17] Watts N, Amann M, Arnell N, Ayeb-Karlsson S, Belesova K, Boykoff M, Byass P, Cai W, Campbell-Lendrum D, Capstick S, Chambers J, Dalin C, Daly M, Dasandi N, Davies M, Drummond P, Dubrow R, Ebi K, Eckelman M, Ekins P, Escobar L, Fernandez Montoya L, Georgeson L, Graham H, Haggar P, Hamilton I, Hartinger S, Hess J, Kelman I, Kiesewetter G, Kjellstrom T, Kniveton D, Lemke B, Liu Y, Lott M, Lowe R, Sewe MO, Martinez-Urtaza J, Maslin M, McAllister L, McGushin A, Jankin Mikhaylov S, Milner J, Moradi-Lakeh M, Morrissey K, Murray K, Munzert S, Nilsson M, Neville T, Oreszczyn T, Owfi F, Pearman O, Pencheon D, Phung D, Pye S, Quinn R, Rabbaniha M, Robinson E, Rocklöv J, Semenza J, Sherman J, Shumake-Guillemot J, Tabatabaei M, Taylor J, Trinanes J, Wilkinson P, Costello A, Gong P, Montgomery H (2019). The 2019 report of The Lancet Countdown on health and climate change: ensuring that the health of a child born today is not defined by a changing climate. Lancet.

[18] Beggs P, Zhang Y, Bambrick H, Berry H, Linnenluecke M, Trueck S, Bi P, Boylan S, Green D, Guo Y, Hanigan I, Johnston F, Madden D, Malik A, Morgan G, Perkins-Kirkpatrick S, Rychetnik L, Stevenson M, Watts N, Capon A (2019). The 2019 report of the MJA–Lancet Countdown on health and climate change: a turbulent year with mixed progress. Med J of Australia.

[19] Zhang Y, Beggs P, Bambrick H, Berry H, Linnenluecke M, Trueck S, Alders R, Bi P, Boylan S, Green D, Guo Y, Hanigan I, Hanna E, Malik A, Morgan G, Stevenson M, Tong S, Watts N, Capon A (2018). The MJA–Lancet Countdown on health and climate change: Australian policy inaction threatens lives. Med J of Australia.

[20] Paterson J, Ford J, Ford L, Lesnikowski A, Berry P, Henderson J, Heymann J (2012). Adaptation to climate change in the Ontario public health sector. BMC Public Health.

[21] Hess J, McDowell J, Luber G (2012). Integrating climate change adaptation into public health practice: using adaptive management to increase adaptive capacity and build resilience. Environ Health Perspect.

[22] World Health Organization (2015). Operational framework for building climate resilient health systems.

[23] Lauriola P, Crabbe H, Behbod B, Yip F, Medina S, Semenza J, Vardoulakis S, Kass D, Zeka A, Khonelidze I, Ashworth M, de Hoogh K, Shi X, Staatsen B, Knudsen L, Fletcher T, Houthuijs D, Leonardi G (2020). Advancing global health through environmental and public health tracking. Int J Environ Res Public Health.

[24] Whittemore R, Knafl K (2005). The integrative review: updated methodology. J of Advanced Nursing.

[25] Toronto C, Remington R (2020). A step-by-step guide to conducting an integrative review.

[26] Moola S, Munn Z, Sears K, Sfetcu R, Currie M, Lisy K, Tufanaru C, Qureshi R, Mattis P, Mu P (2015). Conducting systematic reviews of association (etiology) The Joanna Briggs Institute’s approach. Int J of Evidence-Based Healthcare.

[27] Haddaway N, Collins A, Coughlin D, Kirk S (2015). The role of google scholar in evidence reviews and its applicability to grey literature searching. PLoS ONE.

[28] Moher D, Liberati A, Tetzlaff J, Altman D (2009). Preferred reporting items for systematic reviews and meta-analyses: the PRISMA statement. Phys Ther.

[29] McArthur A, Klugarova J, Yan H & Florescu S. Systematic reviews of text and opinion. In: E Aromataris & Z Munn, editors. JBI Manual for Evidence Synthesis. Adelaide: Joanna Briggs Institute; 2020. 134–174. 10.46658/JBIMES-20-05

[30] Aitsi-Selmi A, Murray V (2015). Protecting the health and well-being of populations from disasters: health and health care in the Sendai Framework for Disaster Risk Reduction 2015–2030. Prehospital & Disaster Med.

[31] Banwell N, Rutherford S, Mackey B, Chu C (2018). Towards improved linkage of disaster risk reduction and climate change adaptation in health: a review. Int J Environ Res & Public Health.

[32] Banwell N, Rutherford S, Mackey B, Street R, Chu C (2018). Commonalities between disaster and climate change risks for health: a theoretical framework. Int J Environ Res & Public Health.

[33] Berry P, Enright P, Shumake-Guillemot J, Villalobos Prats E, Campbell-Lendrum D (2018). Assessing health vulnerabilities and adaptation to climate change: a review of international progress. Int J Environ Res & Public Health.

[34] Ebi K, Vanos J, Baldwin J, Bell J, Hondula D, Errett N, Hayes K, Reid C, Saha S, Spector J, Berry P (2021). Extreme weather and climate change: population health and health system implications. Ann Rev of Public Health.

[35] Keim M, Lemery J, Knowlton K, Sorensen C (2021). Climate-related disasters: the role of prevention for managing health risk. Global climate change and human health: from science to practice.

[36] Aitsi-Selmi A, Wannous C & Murray V. Health supporting disaster risk reduction including climate change adaptation In: I Kelman, J Mercer & J Gaillard, editors. The routledge handbook of disaster risk reduction including climate change adaptation. London: Routledge; 2017. 469–480. 10.4324/9781315684260

[37] Fox M, Zuidema C, Bauman B, Burke T, Sheehan M (2019). Integrating public health into climate change policy and planning: state of practice update. Int J Environ Res & Public Health.

[38] Schramm P, Ahmed M, Siegel H, Donatuto J, Campbell L, Raab K, Svendsen E (2020). Climate change and health: local solutions to local challenges. Current Environ Health Reports.

[39] Sheehan M, Fox M, Kaye C, Resnick B (2017). Integrating health into local climate response: lessons from the U.S CDC climate-ready states and cities initiative. Environ Health Perspect..

[40] World Health Organization. WHO guidance to protect health from climate change through health adaptation planning Geneva: WHO; 2014. https://iris.who.int/bitstream/handle/10665/137383/9789241508001_eng.pdf?sequence=1.

[41] World Health Organization (2021). Climate change and health vulnerability and adaptation assessment.

[42] United Nations Office for Disaster Risk Reduction (2022). Health emergency and disaster risk management: an emerging framework for achieving synergies among the Sendai Framework, the 2030 Agenda for Sustainable Development, the New Urban Agenda and the Paris Agreement.

[43] World Health Organization (2019). Health emergency and disaster risk management framework.

[44] Frumkin H, Hess J, Luber G, Malilay J, McGeehin M (2008). Climate change: the public health response. Am J of Public Health.

[45] Bowen K, Friel S (2012). Climate change adaptation: where does global health fit in the agenda?. Global Health.

[46] Greenberg H (2022). An inflection point in global public health. Global Health.

[47] Government of Western Australia. Health risk assessment. n.d. https://health.wa.gov.au/Articles/F_I/Health-risk-assessment. Accessed 28 October 2023.

[48] United Nations Office for Disaster Risk Reduction. E. health aspect in disaster risk assessment. Geneva: UNDRR; 2017. https://www.preventionweb.net/files/52828_ehealthaspect%5B1%5D.pdf.

[49] Ebi K, Semenza J, Rocklöv J (2016). Current medical research funding and frameworks are insufficient to address the health risks of global environmental change. Environ Health.

[50] Boylan S, Beyer K, Schlosberg D, Mortimer A, Hime N, Scalley B, Alders R, Corvalan C, Capon A (2018). A conceptual framework for climate change, health and wellbeing in NSW. Australia. Public Health Res Prac.

[51] Hambling T, Weinstein P, Slaney D (2011). A review of frameworks for developing environmental health indicators for climate change and health. Int J Environ Res & Public Health.

[52] World Health Organization (2013). Protecting health from climate change: vulnerability and adaptation assessment.

[53] Waheed B, Khan F & Veitch B. Linkage-based frameworks for sustainability assessment: making a case for driving force-pressure-state-exposure-effect-action (DPSEEA) frameworks. Sustainability. 2009;1(3):441–63. https://10.3390/su1030441

[54] Briggs D (2003). Making a difference: indicators to improve children’s environmental health.

[55] World Health Organization. Monitoring health impacts of climate change meeting report. EUR/01/502 6360. Copenhagen: WHO Regional Office for Europe; 2001. https://www.pik-potsdam.de/en/output/publications/pikreports/.files/pr91.pdf.

[56] Füssel H & Klein R. Conceptual frameworks of adaptation to climate change and their applicability to human health. Postdam: Potsdam Institute for Climate Impact Research; 2004. https://www.pik-potsdam.de/en/output/publications/pikreports/.files/pr91.pdf.

[57] World Health Organization. Gothenburg consensus paper - health impact assessment: main concepts and suggested approach. Copenhagen WHO Regional Office for Europe; 1999. http://www.healthedpartners.org/ceu/hia/hia01/01_02_gothenburg_paper_on_hia_1999.pdf.

[58] Tonmoy F, Cooke S, Armstrong F, Rissik D (2020). From science to policy: development of a climate change adaptation plan for the health and well-being sector in Queensland. Australia Environ Science & Policy.

[59] Brown H, Proust K, Spickett J, Capon A (2018). The potential role of health impact assessment in tackling the complexity of climate change adaptation for health. Health Prom J of Australia.

[60] World Health Organization (2022). Compendium of WHO and other UN guidance on health and environment.

[61] Spickett J, Brown H & Katscherian D. Health impacts of climate change: adaptation strategies for Western Australia. Western Australia: Department of Health; 2008. https://www.health.wa.gov.au/~/media/Files/Corporate/general%20documents/Environmental%20health/Climate%20change/Health-impacts-of-climate-change.pdf.

[62] Remais J, Hess J, Ebi K, Markandya A, Balbus J, Wilkinson P, Haines A, Chalabi Z (2014). Estimating the health effects of greenhouse gas mitigation strategies: addressing parametric, model, and valuation challenges. Environ Health Perspect.

[63] Howes M, Tangney P, Reis K, Grant-Smith D, Heazle M, Bosomworth K, Burton P (2015). Towards networked governance: improving interagency communication and collaboration for disaster risk management and climate change adaptation in Australia. J of Enviro Planning and Management.

[64] Preston B & Stafford-Smith M. Framing vulnerability and adaptive capacity assessment: discussion paper. CSIRO climate adaptation flagship working paper no. 2. Canberra: CSIRO; 2009. https://research.csiro.au/climate/wp-content/uploads/sites/54/2016/03/2_Working-Paper2_CAF_PDFStandard.pdf.

[65] Harris P, Spickett J (2011). Health impact assessment in Australia: a review and directions for progress. Environ Impact Assess Review.

[66] Haigh F, Harris E, Chok H, Baum F, Harris-Roxas B, Kemp L, Spickett J, Keleher H, Morgan R, Harris M, Wendel A, Dannenberg A (2013). Characteristics of health impact assessments reported in Australia and New Zealand 2005–2009. Aust & NZ J of Public Health.

[67] Murray C, Ezzati M, Lopez A, Rogers A, Vander HS, Ezzati M, Lopez A, Rogers A, Murray C (2004). Comparative quantification of health risks: conceptul framework and methodological issues. Comparative quantification of health risks global and regional burden of disease attributable to selected major risk factors.

[68] Campbell-Lendrum D, Woodruff R (2006). Comparative risk assessment of the burden of disease from climate change. Environ Health Perspect.

[69] Murray C, Lopez A (2017). Measuring global health: motivation and evolution of the global burden of disease study. Lancet.

[70] Plass D, Hilderink H, Lehtomäki H, Øverland S, Eikemo T, Lai T, Gorasso V, Devleesschauwer B (2022). Estimating risk factor attributable burden: challenges and potential solutions when using the comparative risk assessment methodology. Arch public health.

[71] Watts N, Amann M, Ayeb-Karlsson S, Belesova K, Bouley T, Boykoff M, Byass P, Cai W, Campbell-Lendrum D, Chambers J, Cox P, Daly M, Dasandi N, Davies M, Depledge M, Depoux A, Dominguez-Salas P, Drummond P, Ekins P, Flahault A, Frumkin H, Georgeson L, Ghanei M, Grace D, Graham H, Grojsman R, Haines A, Hamilton I, Hartinger S, Johnson A, Kelman I, Kiesewetter G, Kniveton D, Liang L, Lott M, Lowe R, Mace G, Odhiambo Sewe M, Maslin M, Mikhaylov S, Milner J, Latifi A, Moradi-Lakeh M, Morrissey K, Murray K, Neville T, Nilsson M, Oreszczyn T, Owfi F, Pencheon D, Pye S, Rabbaniha M, Robinson E, Rocklöv J, Schütte S, Shumake-Guillemot J, Steinbach R, Tabatabaei M, Wheeler N, Wilkinson P, Gong P, Montgomery H, Costello A (2018). The Lancet Countdown on health and climate change: from 25 years of inaction to a global transformation for public health. Lancet.

[72] Houghton A, English P (2014). An approach to developing local climate change environmental public health indicators, vulnerability assessments, and projections of future impacts. J of Environ & Public Health.

[73] Ebi K, Boyer C, Bowen K, Frumkin H, Hess J (2018). Monitoring and evaluation indicators for climate change-related health impacts, risks, adaptation, and resilience. Int J Environ Res & Public Health.

[74] Centers for Disease Control and Prevention. Public health systems and best practices. 2023. https://www.cdc.gov/publichealthgateway/bestpractices/index.html. Accessed 7 July 2023.

[75] Downey L, Thomas W, Gaddam R, Scutchfield F (2013). The relationship between local public health agency characteristics and performance of partnership-related essential public health services. Health Prom Prac.

[76] Marinucci G, Luber G, Uejio C, Saha S, Hess J (2014). Building resilience against climate effects: a novel framework to facilitate climate readiness in public health agencies. Int J Environ Res Public Health.

[77] Haasnoot M, Kwakkel J, Walker W, ter Maat J (2013). Dynamic adaptive policy pathways: a method for crafting robust decisions for a deeply uncertain world. Global Eviron Change.

[78] United Nations Framework Convention on Climate Change. National adaptation plans. 2021. https://unfccc.int/topics/adaptation-and-resilience/workstreams/national-adaptation-plans. Accessed 2 July 2023.

[79] Ebi K, Prats E (2015). Health in national climate change adaptation planning. Ann Glob Health.

[80] United Nations Framework Convention on Climate Change (2017). Opportunities and options for integrating climate change adaptation with the Sustainable Development Goals and the Sendai Framework for Disaster Risk Reduction 2015–2030 technical paper by the secretariat.

[81] Australian Government. National climate resilience and adaptation strategy 2021–2025. Canberra: Department of Agriculture, Water and the Environment; 2021. https://www.agriculture.gov.au/sites/default/files/documents/national-climate-resilience-and-adaptationstrategy.pdf.

[82] Armstrong F, Cooke S, Rissik D & Tonmoy F. Human health and well-being climate change adaptation plan for Queensland. Brisbane: Queensland Government; 2018. https://www.qld.gov.au/__data/assets/pdf_file/0022/64237/h-cap-qld.pdf.

[83] Ebi K (2011). Climate change and health risks: assessing and responding to them through ‘adaptive management’. Health Aff.

[84] World Health Organization (2000). The world health report 2000.

[85] Papanicolas I, Papanicolas I, Smith P (2013). International frameworks for health system comparison. Health system performance comparison: an agenda for policy information and research.

[86] Augustynowicz A, Opolski J, Waszkiewicz M (2022). Resilient health and the healthcare system: a few introductory remarks in times of the COVID-19 pandemic. Int J Environ Res Public Health.

[87] European Environment Agency (2017). Climate change adaptation and disaster: enhancing coherence of the knowledge base, policies and practices.

[88] Organisation for Economic Co-Operation and Development (2020). Common ground between the Paris Agreement and the Sendai Framework: climate change adaptation and disaster risk reduction.

[89] Islam S, Chu C, Smart J, Liew L (2020). Integrating disaster risk reduction and climate change adaptation: a systematic literature review. Climate and Develop.

[90] Commonwealth of Australia. National disaster risk reduction framework. Canberra: Department of Home Affairs; 2018. https://www.homeaffairs.gov.au/emergency/files/national-disaster-risk-reduction-framework.pdf.

[91] United Nations Office for Disaster Risk Reduction (2020). Disaster risk reduction in Australia: status report 2020.

[92] Raikes J, Smith T, Baldwin C, Henstra D (2022). Disaster risk reduction and climate policy implementation challenges in Canada and Australia. Climate Policy.

[93] Wright N, Fagan L, Lapitan J, Kayano R, Abrahams J, Huda Q, Murray V (2020). Health emergency and disaster risk management: five years into implementation of the Sendai Framework. Int J of Disaster Risk Science.

[94] Reisinger A, Howden M, Vera C, Garschagen M, Hurlbert M, Kreibiehl S, Mach K, Mintenbeck K, O’Neill B, Pathak M, Pedace R, Pörtner H, Poloczanska E, Rojas Corradi M, Sillmann J, van Aalst M, Viner D, Jones R, Ruane A, Ranasinghe R (2020). The concept of risk in the IPCC sixth assessment report: a summary of cross-working group discussions.

[95] English P, Richardson M (2016). Components of population vulnerability and their relationship with climate-sensitive health threats. Curr Environ Health Reps.

[96] United Nations Office for Disaster Risk Reduction and International Science Council. Hazard definition and classification review (Technical Report) UNDRR: Geneva ISC: Paris; 2020. https://www.undrr.org/publication/hazard-definition-and-classification-review-technical-report.

[97] Fakhruddin B, Sims B (2021). Analysis of DRR inclusion in national climate change commitments.

[98] International Science Council; United Nations Office for Disaster Risk Reduction; Integrated Research on Disaster Risk. A framework for global science in support of risk informed sustainable development and planetary health. ISC: Paris, UNDRR: Geneva, IRD: Beijing; 2021. 10.24948/2021.07.

[99] Sillmann J, Christensen I, Hochrainer-Stigler S, Huang-Lachmann, J, Juhola S, Kornhuber K, Mahecha M, Mechler R, Reichstein M, Ruane A, Schweizer P & Williams S. Briefing note on systemic risk. Risk Knowledge Action Network International Climate Research: Oslo United Nations Office for Disaster Risk Reduction: Geneva; 2022. https://www.undrr.org/publication/briefing-note-systemic-risk.

[100] United Nations Office for Disaster Risk Reduction. Recommendations for a revised EU strategy on climate change adaptation Beligum: UNDRR 2020. https://www.undrr.org/publication/recommendations-revised-eu-strategy-climate-change-adaptation.

[101] Thibodeaux J (2021). Conceptualizing multilevel research designs of resilience. J of Community Psychol.

[102] Yang X, Lo K (2021). Environmental health research and the COVID-19 pandemic: a turning point towards sustainability. Environ Research.

[103] United Nations Office for Disaster Risk Reduction (2015). Sendai Framework for Disaster Risk Reduction 2015–2030.

[104] Tasri E, Karimi K, Muslim I (2022). The effect of economic variables on natural disasters and the impact of disasters on economic variables. Heliyon.

[105] Plough A, Fielding J, Chandra A, Williams M, Eisenman D, Wells K, Law G, Fogleman S, Magana A (2013). Building community disaster resilience: perspectives from a large urban county department of public health. Am J of Public Health.

[106] United Nations Office for Disaster Risk Reduction (2020). Integrating disaster risk reduction and climate change adaptation in the UN sustainable development cooperation framework.

[107] Nomura S, Parsons A, Hirabayashi M, Kinoshita R, Liao Y, Hodgson S (2016). Social determinants of mid- to long-term disaster impacts on health: a systematic review. Int J of Disaster Risk Reduction.

[108] Institute of Medicine (U.S.) Committee on Post-Disaster Recovery of a Community’s Public Health. Healthy, resilient, and sustainable communities after disasters: strategies, opportunities, and planning for recovery. Washington, DC: The National Academies Press; 2015. https://www.ncbi.nlm.nih.gov/books/NBK316532/. 26401544

[109] Commonwealth of Australia. Health and disaster management handbook. 2nd edn. Canberra: Australian Institute for Disaster Resilience; 2019. https://knowledge.aidr.org.au/resources/health-and-disaster-management-handbook/.

[110] Glasser R. Preparing for the era of disasters. Canberra: Australian Strategic Policy Institute; 2019. https://www.jstor.org/stable/pdf/resrep23057.1.pdf.

[111] Dasandi N, Graham H, Lampard P, Jankin MS (2021). Intergovernmental engagement on health impacts of climate change. Bull World Health Organ.

[112] Shiell A, Jackson H (2018). How much does Australia spend on prevention and how would we know whether it is enough?. Health Prom J of Australia.

[113] Wright M, Versteeg R, van Gool K (2021). How much of Australia’s health expenditure is allocated to general practice and primary healthcare?. Australian J Gen Practice.

[114] Organisation for Economic Co-Operation and Development. Health expenditure and financing 2023. https://stats.oecd.org/Index.aspx?DataSetCode=SHA. Accessed 7 July 2023.

[115] Smith J, Herriot M, Williams C, Judd J, Griffiths K, Bainbridge R (2019). Health promotion: a political imperative. Health Prom J of Australia.

[116] Taylor R, Sullivan D, Reeves P, Kerr N, Sawyer A, Schwartzkoff E, Bailey A, Williams C, Hure A (2023). A scoping review of economic evaluations to inform the reorientation of preventive health services in Australia. Int J of Environ Res & Public Health.

[117] Johnston I (2020). Australia’s public health response to COVID-19: what have we done, and where to from here?. Aust & NZ J of Public Health.

[118] Australia Government. National health and climate strategy. 2023. https://www.health.gov.au/our-work/national-health-and-climate-strategy. Accessed 2 July 2023.

[119] Australia Government. Budget 2023–24: paper no. 2. Canberra: Commonwealth of Australia; 2023. https://budget.gov.au/content/bp2/index.htm.

[120] Eburn M. Coordination of federal, state and local disaster management arrangements in Australia: lessons from the UK and the US. Canberra: The Australian Strategic Policy Institute Limited; 2017. https://www.aspi.org.au/report/coordination-federal-state-and-local-disaster-management-arrangements-australia-lessons-uk.

[121] Horsburgh N, Armstrong F & Mulvenna V. Framework for a national strategy on climate, health and well-being for Australia. Canberra: Climate and Health Alliance; 2017. https://www.caha.org.au/national_strategy_framework_launch.

[122] Smith J, Patrick R (2021). The global climate and health agenda: Australia must do more. Health Prom J of Australia.

[123] Bowen K, Ebi K (2017). Health risks of climate change in the World Health Organization South-East Asia Region. WHO South-East Asia J of Public Health.

[124] World Health Organization (2018). Key learning on health in all policies implementation from around the world – information brochure.

[125] Macassa G, Ribeiro A, Marttila A, Stål F, Silva J, Rydback M, Rashid M, Barros H (2022). Public health aspects of climate change adaptation in three cities: a qualitative study. Int J Environ Res Public Health.

[126] Patz J, Thomson M (2018). Climate change and health: moving from theory to practice. PLoS Med.

[127] Rudolph L, Caplan J, Ben-Moshe K, Dillon L (2013). Health in all policies: a guide for state and local governments.

[128] Kendrovski V, Schmoll O (2019). Priorities for protecting health from climate change in the WHO European Region: recent regional activities. Bundesgesundheitsbl.

