# Supplementary material for: Climate Change Adaptation Methods for Public Health Prevention in Australia: an Integrative Review
Source: Curr Environ Health Rep. 2024 Jan 15;11(1):71–87. doi: 10.1007/s40572-023-00422-7 (PMC10907446; doi:10.1007/s40572-023-00422-7)
Supplement: Supplementary file 1 — Supplementary file1 (DOCX 25.7 KB) [file 40572_2023_422_MOESM1_ESM.docx]

**Attachment 1 – Search strategy.**

Web of Science Search

| 1 | ALL=("public health") |
| --- | --- |
| 2 | ALL=("environmental health") |
| 3 | **#1 OR #2** |
| 4 | ALL=("climate change" ) |
| 5 | ALL=("disaster" ) |
| 6 | ALL=("natural hazard" ) |
| 7 | ALL=("extreme weather" ) |
| 8 | ALL=("vulnerabl*") |
| 9 | **#8 OR #7 OR #6 OR #5 OR #4** |
| 10 | ALL=("climate change adapt*") |
| 11 | ALL=("adaptive capacity") |
| 12 | ALL=("resilien*") |
| 13 | ALL=("disaster risk reduction") |
| 14 | ALL=("prevent*") |
| 15 | **#14 OR #13 OR #12 OR #11 OR #10** |
| 16 | **#15 AND #9 AND #3** |
| 17 | **Limit 16 to (year = “2012 – 2022”) and (Languages = English) and Exclude Letter or Book Chapters or Meeting Abstract or Correction or Book Review or Data Paper or News Item or Publication With Expression Of Concern or Retracted Publication or Editorial Material or Early Access or Proceeding Paper or Article)** |

CINAHL

| 1 | ALL=("public health") |
| --- | --- |
| 2 | ALL=("environmental health") |
| 3 | **#1 OR #2** |
| 4 | ALL=("climate change" ) |
| 5 | ALL=("disaster" ) |
| 6 | ALL=("natural hazard" ) |
| 7 | ALL=("extreme weather" ) |
| 8 | ALL=("vulnerabl*") |
| 9 | **#8 OR #7 OR #6 OR #5 OR #4** |
| 10 | ALL=("climate change adapt*") |
| 11 | ALL=("adaptive capacity") |
| 12 | ALL=("resilien*") |
| 13 | ALL=("disaster risk reduction") |
| 14 | ALL=("prevent*") |
| 15 | **#14 OR #13 OR #12 OR #11 OR #10** |
| 16 | **#15 AND #9 AND #3** |
| 17 | **Limit 16 to (year = “2012 – 2022”) and (Languages = English) and Exclude Letter or Book Chapters or Meeting Abstract or Correction or Book Review or Data Paper or News Item or Publication With Expression Of Concern or Retracted Publication or Editorial Material or Early Access or Proceeding Paper or Article)** |

MEDLINE *Mesh terms

| 1 | ALL=("public health") *Public Health |
| --- | --- |
| 2 | ALL=("environmental health") *"Social Determinants of Health"/ or *Environmental Health/ |
| 3 | **#1 OR #2** |
| 4 | ALL=("climate change" ) *Climate/ or *Climate Change/ |
| 5 | ALL=("disaster" ) *Natural Disasters/ or *Disasters/ or *Disaster Planning/ |
| 6 | ALL=("natural hazard" ) *Risk Assessment/ or *Floods/ |
| 7 | ALL=("extreme weather" ) *Extreme Weather/ or *Weather/ |
| 8 | ALL=("vulnerabl*") *Vulnerable Populations/ |
| 9 | **#8 OR #7 OR #6 OR #5 OR #4** |
| 10 | ALL=("climate change adapt*") *Adaptation, Physiological |
| 11 | ALL=("adaptive capacity") |
| 12 | ALL=("resilien*") *Resilience, Psychological/ |
| 13 | ALL=("disaster risk reduction") *Risk Assessment/ or *Disaster Planning/ or *Floods/ or *Risk Management/ |
| 14 | ALL=("prevent*") |
| 15 | **#14 OR #13 OR #12 OR #11 OR #10** |
| 16 | **#15 AND #9 AND #3** |
| 17 | **Limit 16 to (year = “2012 – 2022”) and (Languages = English) and Exclude Letter or Book Chapters or Meeting Abstract or Correction or Book Review or Data Paper or News Item or Publication With Expression Of Concern or Retracted Publication or Editorial Material or Early Access or Proceeding Paper or Article)** |

PUBMED Mesh terms

| 1 | ALL=("public health") "Public Health"[MeSH Terms] OR "Public Health Practice"[MeSH Terms] OR "Environment and Public Health"[MeSH Terms] |
| --- | --- |
| 2 | ALL=("environmental health") "Environmental Health"[MeSH Terms] |
| 3 | **#1 OR #2** |
| 4 | ALL=("climate change" ) "Climate"[MeSH Major Topic] |
| 5 | ALL=("disaster" ) "Disasters"[MeSH Terms] |
| 6 | ALL=("natural hazard" ) "Natural Disasters"[MeSH Terms] |
| 7 | ALL=("extreme weather" ) "Extreme Weather"[MeSH Terms] OR"Extreme Hot Weather"[MeSH Terms] |
| 8 | ALL=("vulnerabl*") "Social Vulnerability"[MeSH Terms] |
| 9 | **#8 OR #7 OR #6 OR #5 OR #4** |
| 10 | ALL=("climate change adapt*") "adaptation, psychological"[MeSH Terms] OR "adaptation, physiological"[MeSH Terms] |
| 11 | ALL=("adaptive capacity") |
| 12 | ALL=("resilien*") "resilience, psychological"[MeSH Terms] |
| 13 | ALL=("disaster risk reduction") |
| 14 | ALL=("prevent*") "Preventive Medicine"[MeSH Terms] |
| 15 | **#14 OR #13 OR #12 OR #11 OR #10** |
| 16 | **#15 AND #9 AND #3** |
| 17 | **Limit 16 to (year = “2012 – 2022”) and (Languages = English) and Exclude Letter or Book Chapters or Meeting Abstract or Correction or Book Review or Data Paper or News Item or Publication With Expression Of Concern or Retracted Publication or Editorial Material or Early Access or Proceeding Paper or Article)**  - |

Google Scholar search string date range limited 2012-2022 and first 200 results only

|public health environmental health||climate change natural hazard extreme weather disaster vulnerable||climate change adaptation adaptive capacity resilience disaster risk reduction|

**Attachment 2 – Summary of included articles.**

| **Author/s** | **Title** | **Context** | **Methodology** | **Tool/framework** | **Quality** | **Key Messages** |
| --- | --- | --- | --- | --- | --- | --- |
| Aitsi-Selmi & Murray (2015) | Protecting the Health and Well-being of Populations from Disasters: Health and Health Care in The Sendai Framework for Disaster Risk Reduction 2015-2030 | Global | Report/commentary | Disaster Risk Reduction | Good | Sendai Framework for Disaster Risk Reduction is widening of health sector activities to align with global public health needs |
| Banwell et al. (2018) | Commonalities between Disaster and Climate Change Risks for Health: A Theoretical Framework | Global | Report/commentary | Disaster Risk Reduction | Good | Disaster health impacts share commonalities with health impacts of climate change and have considerable overlaps in the different pathways through which these impacts occur. It is also reasonable to assume that the strategies in place, or being developed, to reduce these risks must also have numerous commonalities for both approaches. |
| Banwell et al. (2018) | Towards Improved Linkage of Disaster Risk Reduction and Climate Change Adaptation in Health: A Review | Global | Review | Disaster Risk Reduction | Good | Linking Disaster Risk Reduction and Climate Change Adaptation is essential for addressing these ever present, complex and increasing risks in health. This is fundamental to maximise conceptual linkages in order to reach common goals including building resilience and reducing risk and vulnerability. Public health actors have a central role in building these links. |
| Berry et al. (2018) | Assessing Health Vulnerabilities and Adaptation to Climate Change: A Review of International Progress | Global | Review | Adaptation and Vulnerability Assessments | Good | Examines the evolution of climate change and health vulnerability and adaptation assessments and described the benefits and limitations identified for countries undertaking these assessments. |
| Delpla et al (2021) | Tools and Methods to Include Health in Climate Change Adaptation and Mitigation Strategies and Policies: A Scoping Review | Global | Review | Health Impact Assessment,  Comparative Risk Assessment,  Adaptation and Vulnerability Assessments,  National Adaptation  Plan,  Driver, Pressure, State, Exposure, Effect, Action Framework | Good | A scoping review to identify tools and methods that help integrate health into climate change adaptation and mitigation policies and strategies. |
| Ebi et al. (2021) | Extreme Weather and Climate Change: Population Health and Health System Implications | Global | Report/commentary | Vulnerability and Adaptation Assessments,  Disaster Risk Reduction | Good | Assessments of population health and health system vulnerabilities and capacities and help evaluate the effectiveness of integrated disaster risk management and adaptation strategies. A key action to close the health adaptation gap is to integrate disaster risk management into all health policies and to integrate health into disaster risk management plans and strategies. |
| Fox et al. (2019) | Integrating Public Health into Climate Change Policy and Planning: State of Practice Update | United States | Review | Building Resilience Against Climate Effects, Health Impact Assessment | Good | Overview of public health over the last decade in climate change policy and tools available with an aim to identify evidence of forward policy and practice movement. An updated core functions and essential services model with strong governance, implementation and dynamic adjustment activities will ensure that the public health field fulfills its potential as a highly valued and proactive partner in climate policy planning and action. |
| Hess, McDowell & Luber  (2012) | Integrating Climate Change Adaptation into Public Health Practice: Using Adaptive Management to Increase Adaptive Capacity and Build Resilience | Global | Review | Adaptive Management | Good | Identify strategies for expanding public health’s adaptive capacity through an emphasis on learning and changes in management frameworks. Iterative method of adaptive management and embracing learning has been suggested to assist in decision making in environments hampered by complexity with a host of management challenges around climate change adaptation. |
| Keim  (2021) | Climate-related disasters: the role of prevention for managing health risk | Global | Report/commentary | Public Health Preparedness | Good | Disaster response and recovery alone is not sufficient. The importance to recognize the value of a comprehensive approach to the preventative health in managing disaster-related health risks. |
| Aitsi-Selmi, Wannous & Murray  (2017) | Health Supporting Disaster Risk Reduction Including Climate Change Adaptation | Global | Report/commentary | Disaster Risk Reduction | Good | Health and human well-being are an element of synergy and point of convergence for the post-2015 agenda, especially for Disaster Risk Reduction Including Climate Change Adaptation. The need to recognise and articulate synergies between the different policy agendas through a health lens. |
| Schramm (2020) | Climate Change and Health: Local Solutions to Local Challenges | United States | Report/commentary | Building Resilience Against Climate Effects | Good | Local level adaption with a focus Building Resilience Against Climate Effects framework and barriers exist that can prevent the development of local solutions. |
| Sheehan et al (2017) | Integrating Health into Local Climate Response: Lessons from the U.S. CDC Climate-Ready States and Cities Initiative | United States | Report/commentary | Building Resilience Against Climate Effects | Good | Local governments form the backbone of climate-related public health preparedness but are often inadequately prepared and poorly integrated into climate change assessments and plans. Building Resilience Against Climate Effects framework offers collaboration as a solution public health departments. |
| United Nations of Disaster Risk Reduction  (2022) | Health Emergency and Disaster Risk Management: An emerging framework for achieving synergies among the Sendai Framework, the 2030 Agenda for Sustainable Development, the New Urban Agenda and the Paris Agreement | Global | Report/commentary | Health Emergency and Disaster Risk  Management | Good | Health Emergency and Disaster Risk Management Framework has a potential in promoting synergies in pursing risk-resilient sustainable development pathways via conceptual analysis of the key roles of health. Four key international risk-resilient and sustainable development agendas are analysed in detail to explore how they can be interlinked under this framework. |
| Wheeler & Watts  (2018) | Climate Change: From Science to Practice | Global | Report/commentary | Building Resilience Against Climate Effects,  adaptive management,  Public Health Preparedness | Good | Building Resilience Against Climate Effects framework provides an excellent approach for health adaptation to climate change. Modified version to include an ‘adaptation pathways approach’ has been suggested. Traditional public health services—such as the distinction of primary, secondary, and tertiary prevention can be helpfully applied to climate change adaptation. |
| World Health Organization  (2015) | Operational framework for building climate resilient health systems | Global | Report/commentary | Operational framework for building climate resilient health systems | Good | The framework provides guidance on how the health sector and its operational basis in health systems can systematically and effectively address the challenges of climate change. By implementing the 10 key components laid out in this framework, health organizations, authorities and programmes will be better able to anticipate, prevent, prepare for and manage climate-related health risks. |
| World Health Organization  (2014) | WHO guidance to protect health from climate change through health adaptation planning | Global | Report/commentary | National Adaptation Plan | Good | This guidance is designed to ensure that the process of iteratively managing the health risks of climate change and health sector is properly represented into the overall National Adaptation Plan process. |
| World Health Organization  (2019) | Health Emergency and Disaster Risk Management Framework | Global | Report/commentary | Health Emergency and Disaster Risk  Management | Good | Reducing the health risks and consequences of emergencies is vital to local to global health security and to build the resilience of communities and health systems. Fragmented approaches, including over-emphasis on reacting to, instead of preventing events and preparing properly, poor coordination within and outside the health system, have hindered the ability to achieve optimal development outcomes including for public health. This framework been developed to consolidate contemporary approaches and practice to scale up risk-informed actions to reduce hazards, exposures and vulnerabilities, and build capacities. |
| World Health Organization  (2021) | Climate change and health vulnerability and adaptation assessment | Global | Report/commentary | Vulnerability and Adaptation Assessment | Good | Vulnerability and adaptation assessment  tool allows countries to evaluate which populations and specific geographies are most vulnerable to different kinds of health effects from climate change; identify weaknesses in the systems and to specify interventions to respond. |
| World Health Organization  (2021) | Quality criteria for health national adaptation plans | Global | Report/commentary | National Adaptation Plan | Good | Health National Adaptation Plan development is critical for: ensuring prioritization of action to address the health impacts of climate all levels of planning; linking the health sector to national and international climate change agendas, including an increased emphasis on health cobenefits of mitigation and adaptation actions in other sectors; promoting and facilitating coordinated and inclusive climate change and health planning among health stakeholders at different levels of government and across health- determining sectors; and enhancing health sector access to climate funding. |
